# Asymmetric AZA-BODIPY with Optical Gain in the Near-Infrared Region

**DOI:** 10.3390/molecules27144538

**Published:** 2022-07-15

**Authors:** Tersilla Virgili, Lucia Ganzer, Chiara Botta, Benedetta Maria Squeo, Mariacecilia Pasini

**Affiliations:** 1 Institute for Photonics and Nanotechnologies (IFN), National Research Council-CNR, Piazza Leonardo da Vinci 32, 20133 Milan, Italy; lucia.ganzer@polimi.it; 2 Istituto di Scienza e Tecnologie Chimiche (SCITEC), National Research Council-CNR, Via Corti, 20133 Milan, Italy; chiara.botta@scitec.cnr.it (C.B.); benedetta.squeo@scitec.cnr.it (B.M.S.)

**Keywords:** AZA-BODIPY, ultrafast spectroscopy, stimulated emission

## Abstract

In recent years, there has been a lot of interest in the development of organic compounds emitting in the near-infrared (NIR) region due to their stimulating applications, such as biosensing and light detection and ranging (LiDAR). Moreover, a lot of effort has been devoted to finding organic emitters with optical gain in the NIR region for lasing applications. In this paper, we present the ultrafast spectroscopy of an asymmetric AZA-BODIPY molecule that shows relevant photophysical changes moving from a diluted solution to a concentrated solution and to a spin-coated film. The diluted solution and the spin-coated film show a bleaching band and a stimulated emission band in the visible region, while the very concentrated solution displays a broad (150 nm) and long-living (more than 400 ps) optical gain band in the NIR region, centered at 900 nm. Our results pave the way for a new organic laser system in a near-infrared spectral region.

## 1. Introduction

Organic optoelectronics and photonics based on the development of molecules and polymers are playing an increasingly important role in biosensing and lasing applications. In fact, in addition to having the characteristics of low cost, easy processability, and high modulation [1,2], they also respond to the request for new sustainable materials, which are necessary for the transition to green technology [3]. In fact, organic materials meet the demand for biocompatibility and low toxicity, and can be obtained from abundant raw materials [4,5].

**Figure 1:**
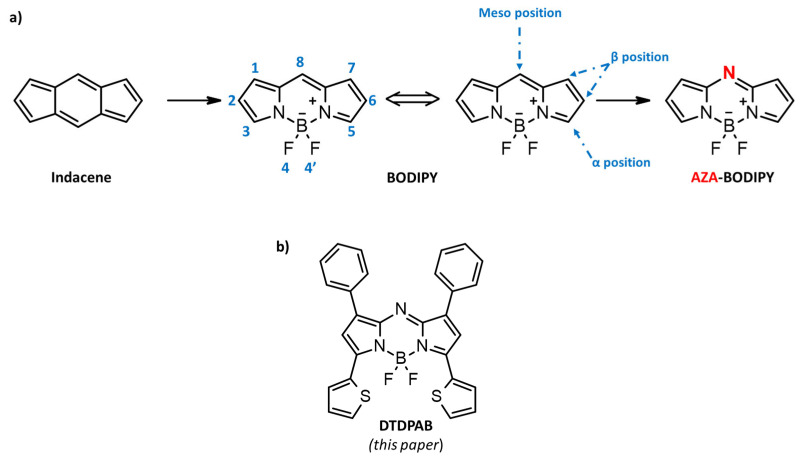
(**a**) From indacene to AZA-BODIPY structure. (**b**) The dithieno-diphenyl-aza-BODIPY (*DTDPAB*) structure.

In recent years, organic semiconductor laser diodes (OSLDs) [6,7] have been intensively studied for the purposes of materials development [8,9,10], mechanism investigation, and device optimization [11]. The use of OSLDs in optoelectronics has many benefits compared to the conventional lasers based on inorganic materials. They are mechanically flexible, easily processed, and their emission wavelength can be easily tuned. In particular, the laser technologies have extended the operating range to 1−2 μm (short-wavelength infrared, SWIR) for their many applications in telecommunications (signal processing, amplification) and in defense (LiDAR, active imaging, telemetry, etc.) [12]. Moreover, in biosensing and healthcare applications [13], OSLDs with emission in the near-infrared (NIR) region from 700 to 900 nm are desirable. Unfortunately, the PL quantum yields (*Φ*_PL_) of NIR emitters are generally very low because of the increase in nonradiative transitions due to the well-established energy gap law [14,15]. As a consequence, it is difficult to find organic material with a high and long-living optical gain in the NIR region. Therefore, the development of materials as well as device architectures with these particular properties has remained a challenge. One of the most promising molecules able to absorb and emit in the 700–900 nm region is 4,4-difluoro-4-bora-3a,4a-diaza-s-indacene (BODIPY) [16,17,18]. BODIPY can be described as a boradiazaindacene on account of the similarity with the all-carbon tricyclic ring having three p-delocalized rings (pyrrole, azafulvene and diazaborinin-type ring) and the numbering of substituents follows rule setup for the carbon polycycles (see Figure 1a).

“BODIPY” might be defined as porphyrin’s “half-brother”, and, in analogy with porphyrinic structures, position 8 is habitually referred as the meso site [19]. The first synthesis of BODIPY was described in 1968 by Treibs and Kreuzer, but the interest of the scientific community in BODIPY has particularly developed in recent years. Its fortune is mainly due to its outstanding photophysical characteristics, such as modulable absorption and emission from the visible to near-infrared (NIR) optical region, combined with high molar extinction coefficients, good fluorescence quantum yields, and excellent photo- and chemical-resistance [20,21,22,23,24,25,26].

From a chemical point of view, BODIPY, thanks to its eight reactive positions, provides a versatile platform for the further functionalization and the consequent fine modulation of optoelectronic and photonic properties [27]. However, the maximum absorption and emission of conventional BODIPY are around 700 nm. In order to red-shift the spectral properties of BODIPY, in 2002 Killoran et al. [28] proposed the replacement of the carbon in position 8, or the meso position, with a nitrogen with the formation of a C=N linkage, which is isoelectronic with the C=CH group but has different geometric and electronic requirements [29,30]. This modification led to the birth of a new family of materials, known as AZA-BODIPY (Figure 1a), that retain the main characteristics of the BODIPY-based molecules, but show a markedly red-shifted absorption and emission and specific linear photophysical and nonlinear optical (NLO) properties [31]. Their use is a subject of great interest for photonics applications, including important multidisciplinary areas such as fluorescence bioimaging [32,33] and organic photovoltaics [34].

In this paper, in order to further shift the chromophore emission in the NIR, we replaced the peripheral positions of AZA-BODIPY with aromatic rings, specifically positions 1 and 7 with aryls, and positions 3 and 5 with thiophenic moieties [35] (see Figure 1b). This new AZA-BODIPY molecule presents an emission in the 700–900 nm region. By ultrafast spectroscopy, we found a large gain region peaking at 900 nm in a concentrated solution. Our results pave the way for a new organic laser system in the near-infrared spectral region.

## 2. Results and Discussion

**Scheme 1:**
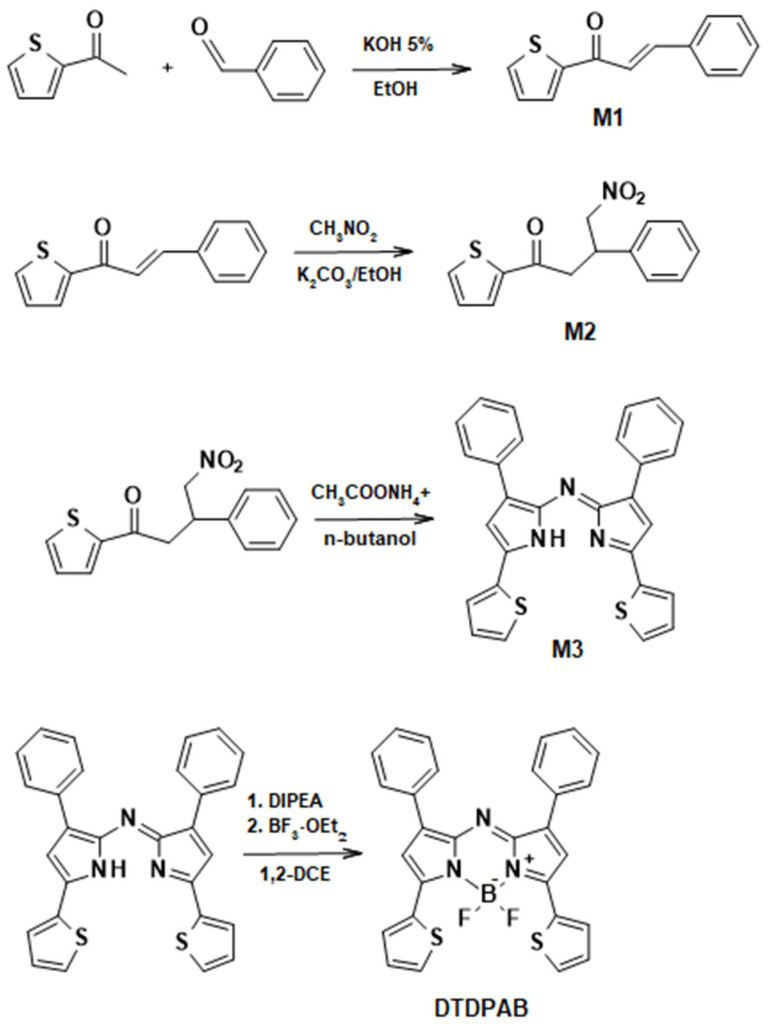
Synthesis of DTDPAB.

The dithieno-diphenyl-aza-BODIPY (*DTDPAB*) is synthetized, according to the literature [35,36], in a four-step reaction, as reported in the experimental section and in Scheme 1. In the first step, the chalcone **M1** is obtained from the condensation between 2-acetylthiophene and benzaldehyde. In the second step, the chalcone reacts quantitatively with nitromethane and potassium carbonate to give the compound phenyl 4-nitro-3-thienylbutan-1-one **M2**, which is used to obtain the azadipyrromethene derivative **M3** after reaction with ammonium acetate. The product dithieno-diphenyl-aza-BODIPY is finally obtained after reacting azadipyrromethene and N,N-diisopropylethylamine with boron trifluoride diethyl etherate.

The presence of a nitrogen atom instead of a carbon atom in the meso position induces the desired red-shift without increasing the molecular size and weight, maintaining a good solubility. The greater electronegativity of nitrogen in the meso position compared to carbon induces a preferable stabilization of the LUMO state, reducing the energy gap. The red-shift can be reinforced through the appropriate lateral substituents, including the aryl rings and electron-rich groups such as the chosen thiophenes, which are able to increase the delocalization (conjugation) with a consequent shift toward the NIR region [37]. The introduction of the thienyl group in positions 3 and 5 has a double effect. It enhances the electron delocalization due to the increased coplanarity, which can be ascribed to the lesser steric hindrance of a five-member ring as compared to the six-member phenyl ring. Contemporarily, the electron-donating thiophene substituent increases the HOMO level, while the LUMO level remains nearly constant, resulting in a reduced gap with a bathochromic shift.

**Figure 2:**
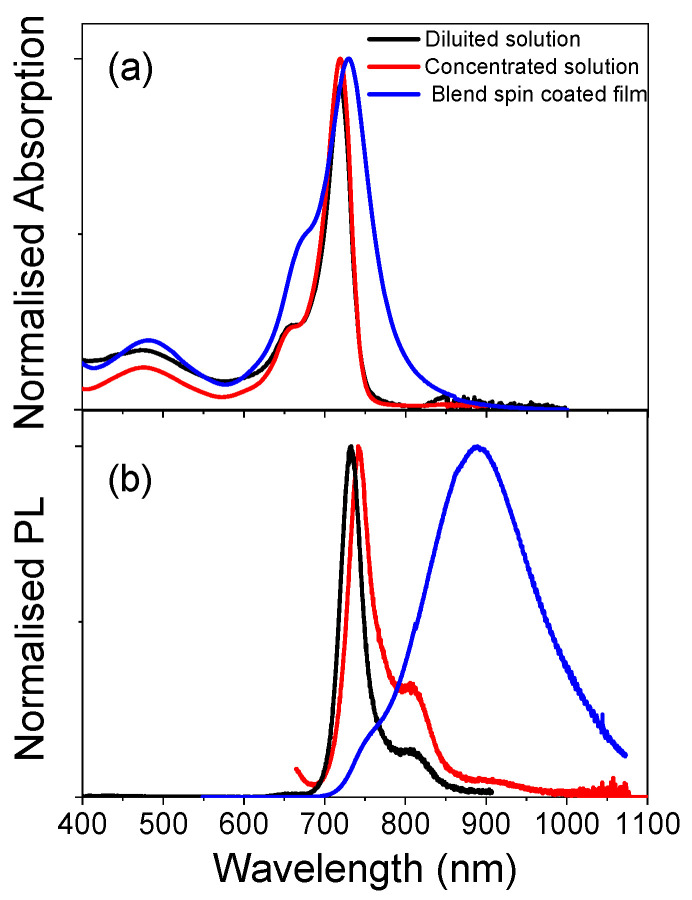
(**a**) Absorption spectra. (**b**) Photoluminescence spectra. Diluted solution (black line), concentrated solution (red line), and spin-coated film using the blend 10% dye in the Polystyrene matrix (blue line).

Figure 2 shows the absorption and the photoluminescence (PL) spectra of the diluted solution, the concentrated solution, and the spin-coated film (10% dye in polystyrene (PS)—see Materials and Methods for details). The absorption spectra of the diluted and concentrated solutions show a main peak at 720 nm with a shoulder at 655 nm. The PL spectra for the two solutions are different. The diluted solution shows a main peak at 735 nm with a shoulder at 810 nm, while the concentrated one shows a 10 nm red-shift of the main peak, with the same shoulder at 810 nm and a new weak band at around 900 nm. The red-shift of the first peak is probably due to self-absorption, due to the small Stokes shift and the high optical density of the concentrated solution. The two shoulders in the PL spectra of the concentrated solution could either both be vibronic replicas of the first singlet state (probably, in the diluted solution, the second band is too weak to be detected), or the 900 nm weak band could be associated with an emission from the aggregates [12]. The spectra of the spin-coated film are quite different from the solution ones.

The absorption spectrum shows a main peak at 730 nm and a shoulder at 670 nm. The main absorption band is much broader (FWHM ≈ 74 nm (160 meV)) than the ones for the two solutions (FWHM ≈ 37 nm (68 meV)). The PL spectrum, then, is dominated by a large band peaked at 900 nm, with a weak shoulder at 750 nm. The broadened absorption spectrum of the film reduces the emission intensity in the 700 nm region due to self-absorption, while the lower energetic band becomes predominant.

We performed transient absorption (TA) measurements of the three samples to elucidate the photophysics of the molecule and to look for optical gain in the NIR region. This technique allowed us to temporally resolve the photophysics of the molecule after photonic excitation. In general, positive differential transmissions are assigned to ground-state photobleaching (GSB) or stimulated emission (SE), while negative features of the TA spectra are attributed to photoinduced absorption (PIA) bands [38]. To distinguish between a GSB and SE signal, we need to look at the absorption and the emission spectra. When the positive transient signal overlaps spectrally with the absorption spectrum, the TA signal is attributed to GSB; otherwise, it is an indication of the presence of stimulated emission, also called optical gain.

**Figure 3:**
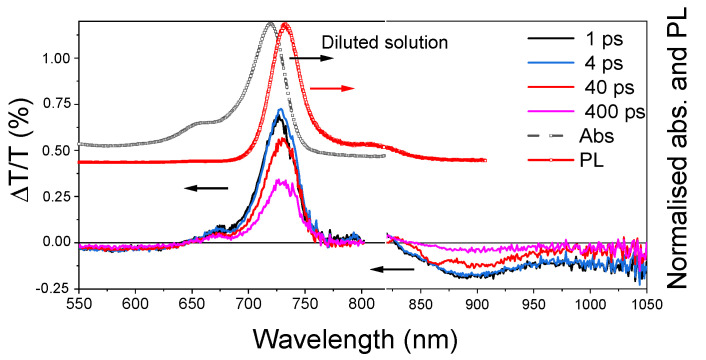
Absorption (red line + symbol) and PL (black line + symbol) spectra (translated vertically). Transient transmission spectra at different probe delays in the visible and NIR spectral regions.

Figure 3 shows the transient transmission spectra of the diluted solution after pumping at 670 nm. The pump–probe spectrum is composed of a positive peak at 725 nm with a shoulder at 660 nm. In the NIR region, a photoinduced absorption band is evident at ≈900 nm. In the figure, the temporal change of the spectrum is shown for the first 400 ps after excitation at delays of 1, 4, 40, and 400 ps after the pump excitation. To better understand these features, the absorption and the PL spectra for the diluted solution are shown in the same plot, translated vertically on the y-axis.

The positive band in the visible region represents the bleaching of the ground state overlapping with the stimulated emission at wavelengths larger than 720 nm. In the NIR region, a broad photoinduced absorption band with a peak at 900 nm is present.

**Figure 4:**
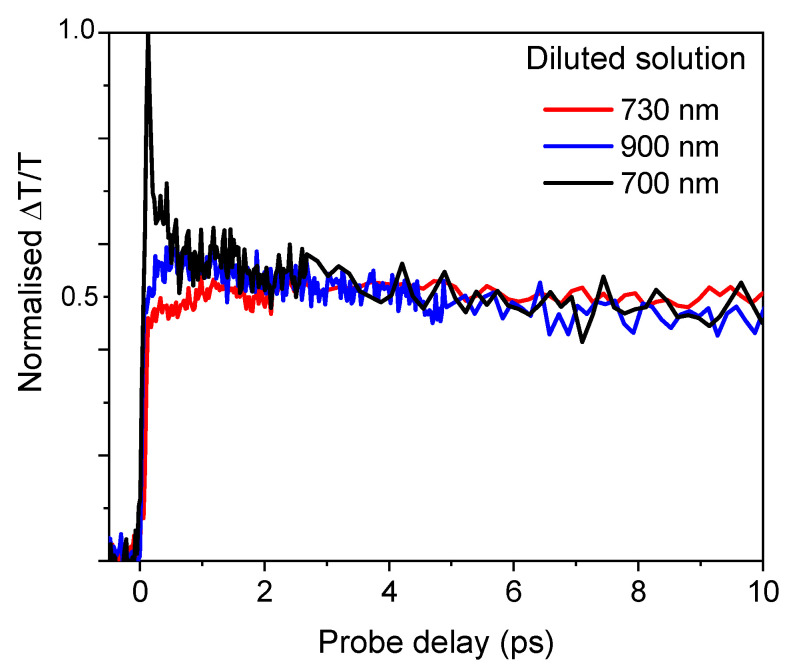
Pump–probe dynamics at different wavelengths.

The temporal dynamics at different wavelengths show a peculiar behavior (see Figure 4), revealing the presence of complex photophysical processes, which are described below. The decay of the bleaching at 700 nm (black line) shows an initial fast component (τ ≈ 1 ps) followed by a very slow one. The 730 nm component (bleaching + stimulated emission, red line) displays a 1ps formation time and then a long-decay component. The decay of the PIA band at 900 nm (blue line) has the same formation time and decay as the 730 nm component. This result indicates that the two main absorption bands at 720 nm and 655 nm (see Figure 2a, black line) can be attributed to S_0_ (singlet ground state)–S_1_ (first singlet electronic state) and S_0_–S_2_ (second singlet electronic state) transitions, respectively. After excitation at 670 nm, the S_2_ (singlet electronic state) is instantaneously populated, and at around 1 ps it decays (see the black line in Figure 4) and populates the S_1_ state. This is revealed by both the dynamics at the 730 nm wavelength (red line in Figure 4), assigned to S_1_–S_0_ stimulated emission, and at 900 nm wavelength (blue line in Figure 4), assigned to the photoinduced S_1_–S_n_ (*n* singlet electronic state) transition. Normally, the energy relaxation from S_2_ to S_1_ is in the order of tens of femtoseconds. Probably, in this case, it is slower due to the different characters of the two singlet states.

In fact, many reports [12,39] show that the singlet states of BODIPY compounds with an AZA-core have a different charge transfer (CT) character. The very strong electron-withdrawing AZA-BODIPY core acts as an accepting trap, preventing complete delocalization across the whole π-conjugated scaffold.

**Figure 5:**
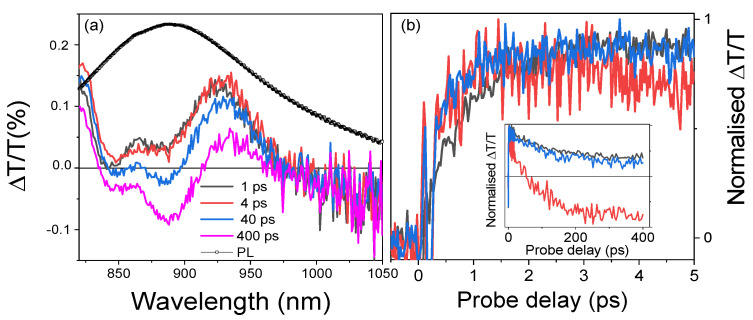
Concentrated solution. (**a**) Transient transmission spectra at different probe delays in the NIR spectral region. The weak PL band is also shown for clarity (grey line with open circles). (**b**) Dynamics at different probe wavelengths: 825 nm (black line), 900 nm (red line), and 930 nm (blue line). In the inset, the same dynamics over a 400 ps temporal window are shown.

Figure 2b shows that by increasing the concentration of the molecule in the solution, a weak band in the NIR region (see the red line) becomes visible in the PL spectrum. To understand the origin of this band, we performed transient transmission measurements in this optical region. The results are shown in Figure 5.

First of all, we note that the spectra of the concentrated solution are completely different from those of the diluted one. After 1 ps from the pump excitation, and until almost 40 ps, the TA signal is positive all across the NIR region between 820–970 nm (see Figure 5a, black, red, and blue lines). Due to the overlap with the broad emission spectrum (Figure 5a, back line + symbols), this signal can be attributed to the presence of optical gain. Unfortunately, after 40 ps, a negative band appears in the region at around 900 nm. Figure 5b shows the temporal decays at different wavelengths. All the temporal traces show a formation time of 2 ps; however, the trace at 900 nm (red line), similarly to the other two in the first 10 ps, becomes negative after 40 ps. This behavior indicates the overlap between a positive signal (SE) and a PIA band.

The analysis of the decays shows that the emissive state is not instantaneously populated. Similar to the diluted solution, in the concentrated ones, the pump excites a higher singlet state, which populates the emissive one. However, for the concentrated solution, a PIA band at 900 nm is also present. This negative contribution lowers and, after 40 ps, completely overcomes the gain in the region between 840 and 920 nm. In any case, the signal remains positive until 400 ps in the other part of the spectrum.

After this good result, we studied the photophysics of the blend 10% *DTDPAB* + PS in the spin-coated thin film. The PL of this film shows a large band centered at 900 nm (Figure 2b, blue line), so we expected to find a large optical region, as in the case of the concentrated solution.

**Figure 6:**
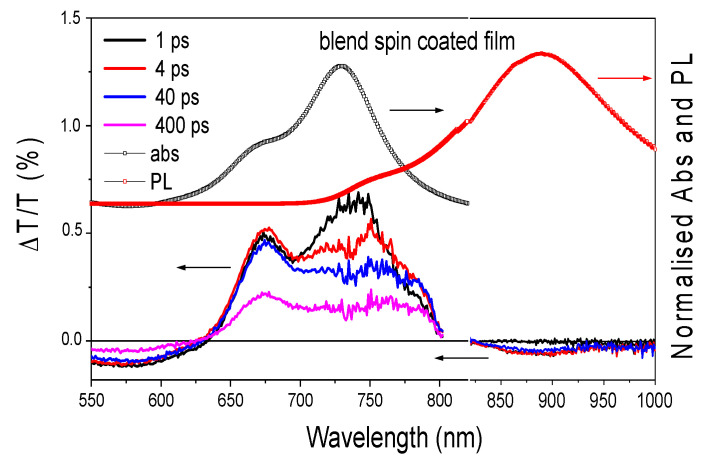
Absorption (black line + symbol) and PL (red line + symbol) spectra (translated vertically). Transient transmission spectra at different probe delays in the visible and NIR spectral regions.

Figure 6 shows the transient transmission spectra of the spin-coated film of the blend 10% dye in PS in the visible and NIR regions. The spectra are composed of a positive peak at 745 nm with a shoulder at 675 nm. In the near-infrared region, a photoinduced absorption band is evident at ≈900 nm and at higher energy with a peak at 580 nm. The photophysics seem to be more similar to those of the diluted solution than those of the concentrated solution. The two positive peaks are attributed to the bleaching of the ground state and the negative PIA band at 900 nm at the S_1_–S_n_ transition.

**Figure 7:**
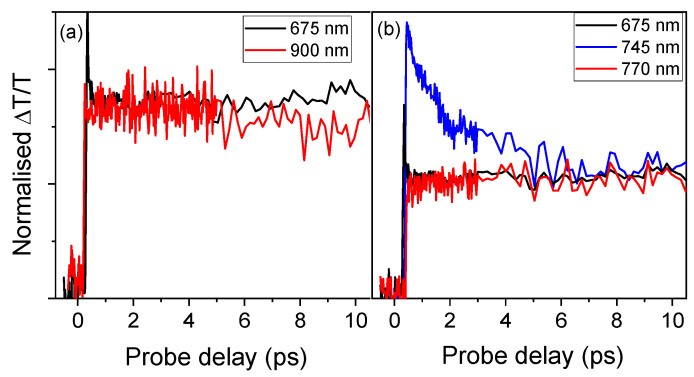
(**a**) Dynamics at different probe wavelengths: 675 nm (black line) and 900 nm (red line). (**b**) Dynamics at different probe wavelengths: 675 nm (black line), 745 nm (blue line), and 770 nm (red line).

However, in this sample, the signal all across the spectral region is instantaneous (see Figure 7), within the 100 fs time resolution of the system, indicating that, with this morphology, the S_2_–S_1_ transition is very fast. Moreover, the bleaching band at 745 nm shows two population contributions: a fast one (τ ≈ 5 ps) and a slow one. Probably, the slow component is the same population responsible for the bleaching at 675 nm and the PIA band at 900 nm, while the fast one is a new population that could be attributed to the aggregated species. More measurements are required to understand the nature of this population. However, unfortunately, it is clear that the stimulated emission in the NIR region that was observed in the concentrated solution is not present in this film.

This behavior could be related to the choice of the matrix. In fact, the BODIPY derivatives are particularly sensitive to the environment that surrounds them, and show a marked solvatochromism [35,40,41]. At this moment, to our knowledge, there are no studies on the effect of polymeric matrices on the optical properties of thin films based on AZA-BODIPY.

## 3. Materials and Methods

*General information for synthesis.* All reagents were purchased from commercial sources, Sigma-Aldrich Chemistry (Schnelldorf, Germany) and TCI (Fukaya, Japan), and were used without further purification. All solvents have been degassed with bubbling nitrogen prior to use. All reactions were carried out in an inert atmosphere.

The 1H-NMR spectra (see Supplementary Materials) were recorded with a Bruker DRX 600 MHz spectrometer (Bruker, Karlsruhe, Germany) and a Bruker ARX 400 MHz spectrometer (Bruker, Karlsruhe, Germany). Gas-phase mass determination was carried out using the Agilent Technologies 7890A GC System (Santa Clara, CA, USA) coupled with an Agilent Technologies 5975C VL MSD (Santa Clara, CA, USA) with a triple-axis mass detector. Elemental analyses were performed on an Elementar Vario EL (Elementar Analysensysteme GmbH, Langenselbold, Germany).

*Synthesis of thienyl-3-phenylprop-2-en-1-one***M1**. A mixture of 2-acetylthiophene (5 g, 39.6 mmol, 1 eq.) and benzaldehyde (4.2 g, 39.6 mmol, 1 eq.) was added to a pre-degassed round-bottom flask, followed by three vacuum/nitrogen cycles. Then, degassed ethanol (200 mL) and potassium hydroxide (KOH) 5% solution (100 mL) in water were added. The mixture was left under stirring at room temperature. After 24 h, a white precipitate was formed. The precipitate was filtered, washed with water, and recrystallized from ethanol (8 g, 94% yield). ^1^H NMR (600 MHz, DMSO) δ 8.35 (d, 1H), 8.06 (d, 1H), 7.89 (m, 3H), 7.73 (d, 1H), 7.47 (m, 3H), 7.32 (m, 1H).

*Synthesis of Phenyl 4-Nitro-3-thienylbutan-1-one***M2**. A mixture of chalcone **M1** (3 g, 14 mmol, 1 eq.), nitromethane (4.27 g, 70 mmol, 5 eq.), and potassium carbonate (K_2_CO_3_, 39 mg, 2% mol) was added to a pre-degassed round-bottom flask, followed by three vacuum/nitrogen cycles. Then, degassed ethanol (14 mL) was added, and the mixture was heated under stirring at reflux for 12 h. After cooling the mixture at room temperature, the solvent was removed under reduced pressure. The crude was then redissolved in ethyl acetate and washed three times with water and brine. The organic phase was then dried over sodium sulfate and the solvent was removed to give a yellowish oil in quantitative yield. The compound was used in the next step without further purification.

*Synthesis of Azadipyrromethene***M3**. A mixture of 4-nitro-3-phenyl-1-(2-thienyl)butan-1-one (**M2**, 3.95 g, 14 mmol, 1 eq.) and ammonium acetate (37.7 mg, 490 mmol, 35 eq.) was dissolved in degassed butanol (185 mL) and left under stirring at reflux for 24 h. After cooling the crude at room temperature, the reaction mixture was diluted with water and extract with dichloromethane and washed three times with brine. The combined organic layer was then dried over sodium sulfate, and the solvent was removed under reduced pressure to give a dark blue solid. The product was purified by silica gel column chromatography, using hexane/chloroform as an eluent to give a crystal powder (480 mg, 8%). ^1^H NMR (600 MHz, CDCl_3_) δ 8.18 (d, 2H), 8.04 (d, 2H), 7.80 (d, 2H), 7.73 (m, 2H), 7.60 (d, 1H), 7.53 (m, 3H), 7.42 (m, 3H), 7.36 (m, 1H), 7.20 (t, 1H), 7.15 (t, 1H), 7.07 (s, 1H).

*Synthesis of DTDPAB.* To a solution of **M3** (200 mg, 0.433 mmol, 1 eq.) in dry dichloroethane we added N,N-diisopropylethylamine (DIPEA, 280 mg, 2.16 mmol, 5 eq.), and the mixture was left under stirring for 1 h. Then, boron trifluoride diethyl etherate (BF_3_·OET_2_) was added, and the mixture was heated to reflux. After 2 h, the mixture was cooled to room temperature and the crude mixture was diluted with dichloromethane and washed with water and brine. The organic layer was dried over magnesium sulphate and the solvent was removed under reduced pressure. The product was purified by silica gel chromatography using hexane/ethyl acetate as an eluent and, subsequently, a further purification by reversed-silica gel chromatography using acetonitrile/methanol (7:3) as an eluent was performed to give an optical-grade purified compound as a coppery, shining powder (180 mg, 81% yield). ^1^H NMR (600 MHz, CDCl_3_) δ 8.41 (d, 2H), 8.08 (d, 2H), 7.66 (d, 2H), 7.47 (m, 6H), 7.30 (t, 2H), 7.21 (s, 2H). MS, (GC-MS) m/z: 509 (M+). Elemental analysis: calculated for C_28_H_18_BF_2_N_3_S_2_: C 66.02, H 3.56, N 8.25, S 12.59; found: C 66.09, H 3.70, N 7.54, S 12.22. The diluted solutions, for measurements, have a concentration of 5 × 10^−5^ M in CHCl_3_, while the concentrated solutions have a concentration of about 5 × 10^−4^ M in CHCl_3._

*Film preparation.* The films were deposited by spin-coating (2000 rpm) from a solution of 3 mg of DTDPAB molecule and 27 mg of polystyrene matrices (10% dye by weight with respect to the polymeric host) dissolved in 0.9 mL of CHCl_3_.

*CW absorption.* The linear absorption spectra were acquired with a JASCO V-750 spectrophotometer (Jasco Europe S.R.L., Cremella (LC), Italy). The measurements were taken in air and at room temperature. The absorption spectra were corrected by the contribution of the UV quartz cuvettes, used for the solutions, and the quartz substrate cuvettes, used for the spin-coated film.

*CW Photoluminescence*. PL spectra are obtained with a NanoLog (Horiba Italia Srl, Milano, Italy) composed by a iH320 spectrograph equipped with a Synapse QExtra charge-coupled device by exciting with a monochromated 450 W Xe lamp. The spectra, obtained by exciting at 650 nm, are corrected for the instrument response.

*Ultrafast TA spectroscopy.* For the pump–probe experiment, we used a Ti: sapphire laser (Libra, Coherent) (Coherent, Santa Clara (CA), USA) characterized by a 100 fs pulse duration, 1 kHz repetition rate, and an 800 nm central wavelength. A pump excitation wavelength of 670 nm was used for the characterization of the samples. The pump light comes from a non-collinear optical parametric amplifier (NOPA). The broadband probe extending in the visible and NIR regions results from white-light continuum generation in a 3 mm-thick sapphire plate pumped by 800 nm light. Using bandpass filters, we selected either the visible range or the NIR range. The delay between the pump and the probe pulses was controlled by a translation stage, and the pump beam was modulated by a mechanical chopper with a 500 Hz frequency. The differential transmission (ΔT/T) of the probe was measured as a function of the probe wavelength and pump–probe delay using an SP2150 Acton spectrometer from Princeton Instruments. The pump energy was adjusted to a fluence of around 10 μJ cm^−2^ for measurements in the visible region and around 45 μJ cm^−2^ for measurements in the NIR region. The measurements were performed with parallel polarizations between the pump and the probe beams.

## 4. Conclusions

In this paper, we synthetized an asymmetric tetra-substituted AZA-BODIPY molecule which displays, in diluted solutions, narrow absorption-emission spectra with a small Stokes shift, red-shifted with respect to BODIPY. A weak emission band at 900nm was detected by increasing the solution concentration. Polystyrene films containing 10% *DTDPAB* displayed a broad NIR emission at 900 nm. Due to the appropriate choice of electron-donor substituents, and to the limited steric hindrance of the thiophenic groups, the dye showed promising gain characteristics in the NIR area.

Transient absorption measurements were performed on the samples. The results for the diluted solutions show ground-state bleaching at 700 nm, overlapping with the stimulated emission at about 730 nm, while in the NIR region, a PIA signal peaking at around 900 nm can be observed. A fast formation time was revealed for the SE signal at 730 nm, suggesting its origin from a higher singlet excited state. The concentrated solution displayed a long-living optical gain in a large (150 nm) NIR optical region (820–970 nm), even if it partly overlapped with the PIA. Unfortunately, we found that an optical gain region in the spin-coated film was absent. This result may be due to the polymeric host used, and it will be the subject of further study. However, the evidence of the presence of a long-living optical gain in concentrated solutions fosters the conditions for the development of a material capable of retaining the same properties, even in the solid state, without being completely quenched by aggregation phenomena.

## Data Availability

The data presented in this study are available on request from the corresponding author. The data are not publicly available due to privacy.

## Supplementary Materials

The following supporting information can be downloaded at: www.mdpi.com/article/10.3390/molecules27144538/s1, Scheme synthesis and ^1^H-NMR of **M1**, **M3**, and DTDPAB.

## Sample Availability

Samples of the compounds used in this researchare available from the authors.
