# Supplementary material for: Asymmetric AZA-BODIPY with Optical Gain in the Near-Infrared Region
Source: Molecules. 2022 Jul 15;27(14):4538. doi: 10.3390/molecules27144538 (PMC9318515; doi:10.3390/molecules27144538)

SUPPLEMENTARY MATERIALS

Asymmetric AZA-BODIPY with Optical Gain in the Near-Infrared Region

Tersilla Virgili [1,*], Lucia Ganzer [1], Chiara Botta [2], Benedetta Maria Squeo [2] and Mariacecilia Pasini [2,*]

1 Institute for Photonics and Nanotechnologies (IFN), National Research Council-CNR, Piazza Leonardo da Vinci 32, 20133 Milan, Italy; lucia.ganzer@polimi.it
2 Istituto di Scienza e Tecnologie Chimiche (SCITEC), National Research Council-CNR, Via Corti, 20133 Milan, Italy; chiara.botta@scitec.cnr.it (C.B.); benedetta.squeo@scitec.cnr.it (B.M.S.)
* Correspondence: tersilla.virgili@polimi.it (T.V.); mariacecilia.pasini@scitec.cnr.it (M.P.)

Scheme synthesis and ^{1}H-NMR of thienyl-3-phenylprop-2-en-1-one.

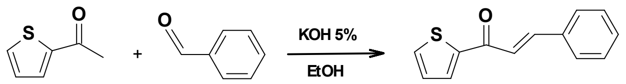

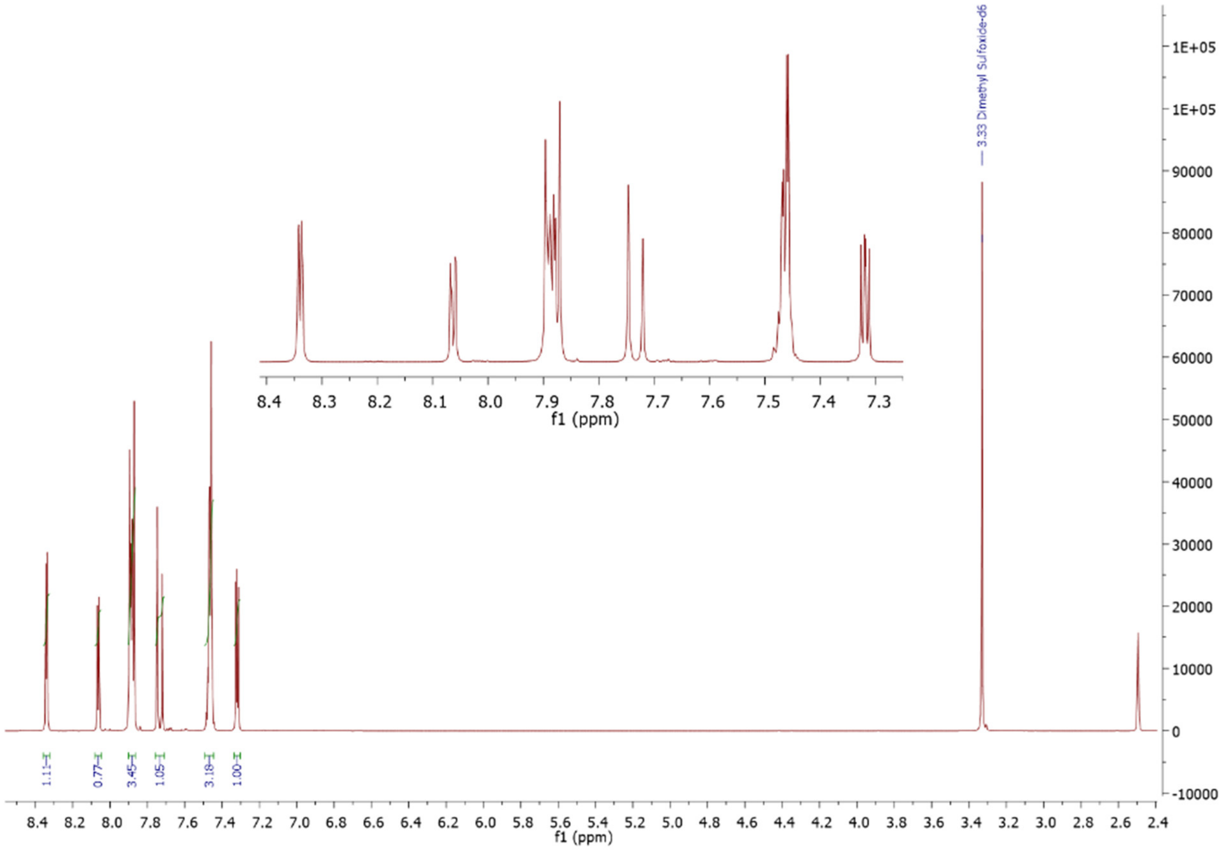

Scheme synthesis of Phenyl 4-Nitro-3-thienylbutan-1-one.

This reaction intermediate was used without further purification in the next reaction and NMR analysis was not performed.

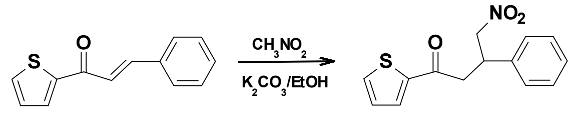

Scheme synthesis and ^{1}H-NMR of Azadipyrromethene.

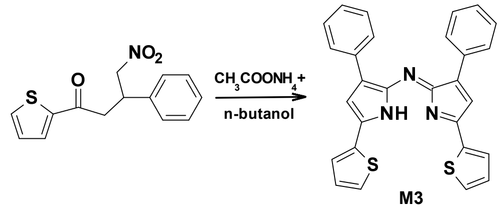

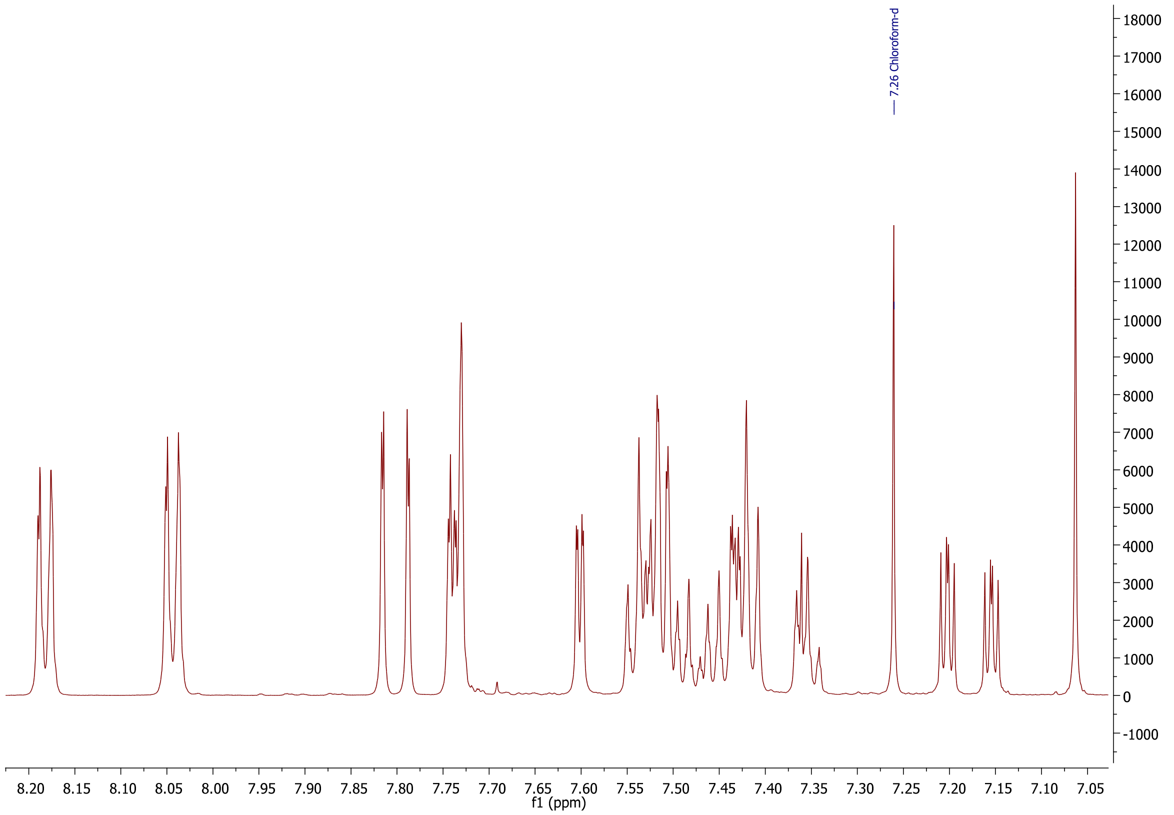

Scheme synthesis and ^{1}H-NMR of DTDPAB.

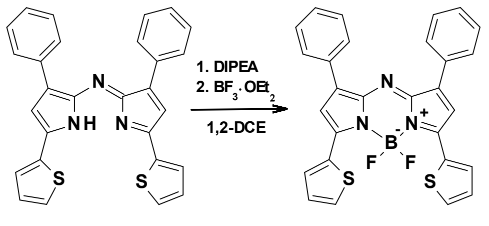

DTDPAB

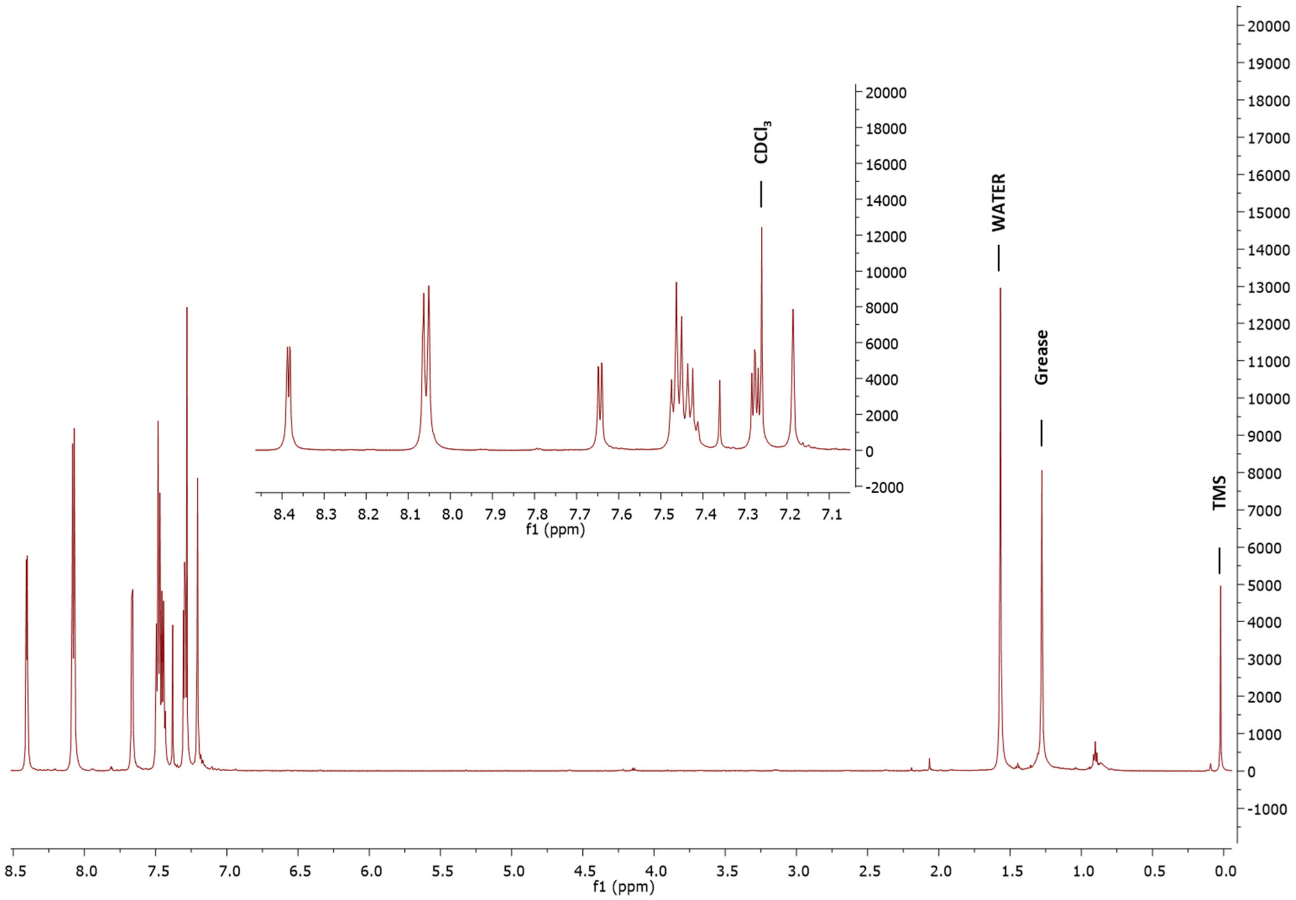

Supplement: Supplementary file 1 [file molecules-27-04538-s001.zip › molecules-1809488-supplementary.pdf]
